# Supplementary material for: Hot spot prediction in protein-protein interactions by an ensemble system
Source: BMC Syst Biol. 2018 Dec 31;12(Suppl 9):132. doi: 10.1186/s12918-018-0665-8 (PMC6311905; doi:10.1186/s12918-018-0665-8)
Supplement: Supplementary file 2 — All relevant MATLAB code. A simple Matlab implement of our predictor. (ZIP 19,742 kb) [file 12918_2018_665_MOESM2_ESM.zip › Additional file 2/Read ASA/ASA Property/SKEMPI/1JCKB.txt]

 NetSurfP - Protein Surface Accessibility and Secondary Structure Predictions

  

|  |  |  |
| --- | --- | --- |
|  |  | NetSurfP - Protein Surface Accessibility and Secondary Structure Predictions  Technical University of Denmark |

  


---

```
# For publication of results, please cite:
# A generic method for assignment of reliability scores applied to solvent accessibility predictions.
# Bent Petersen, Thomas Nordahl Petersen, Pernille Andersen, Morten Nielsen and Claus Lundegaard
# BMC Structural Biology 2009, 9:51 doi:10.1186/1472-6807-9-51
#
# Column 1: Class assignment - B for buried or E for Exposed - Threshold: 25% exposure, but not based on RSA
# Column 2: Amino acid
# Column 3: Sequence name
# Column 4: Amino acid number
# Column 5: Relative Surface Accessibility - RSA
# Column 6: Absolute Surface Accessibility
# Column 7: Z-fit score for RSA prediction
# Column 8: Probability for Alpha-Helix
# Column 9: Probability for Beta-strand
# Column 10: Probability for Coil
E E  1JCKB                  1    0.925 161.615   0.542   0.003   0.003   0.994
E S  1JCKB                  2    0.677  79.356  -0.807   0.058   0.017   0.925
E Q  1JCKB                  3    0.576 102.802  -0.153   0.058   0.017   0.925
E P  1JCKB                  4    0.638  90.532  -0.655   0.058   0.017   0.925
E D  1JCKB                  5    0.595  85.768  -0.480   0.018   0.019   0.964
B P  1JCKB                  6    0.247  35.021  -1.199   0.058   0.017   0.925
E M  1JCKB                  7    0.499  99.810   1.183   0.053   0.005   0.942
E P  1JCKB                  8    0.293  41.591  -0.606   0.406   0.004   0.590
E D  1JCKB                  9    0.712 102.614   0.299   0.522   0.016   0.462
E D  1JCKB                 10    0.428  61.660   0.402   0.522   0.016   0.462
B L  1JCKB                 11    0.088  16.040  -0.072   0.522   0.016   0.462
E H  1JCKB                 12    0.286  52.060   0.348   0.561   0.047   0.393
E K  1JCKB                 13    0.513 105.504   0.358   0.455   0.046   0.498
E S  1JCKB                 14    0.328  38.453  -0.219   0.455   0.046   0.498
B S  1JCKB                 15    0.205  23.967  -1.496   0.354   0.048   0.598
E E  1JCKB                 16    0.524  91.578   0.072   0.354   0.048   0.598
B F  1JCKB                 17    0.181  36.266  -1.108   0.354   0.048   0.598
E T  1JCKB                 18    0.444  61.583  -0.105   0.430   0.016   0.555
E G  1JCKB                 19    0.548  43.143  -1.042   0.522   0.016   0.462
B T  1JCKB                 20    0.213  29.543  -1.095   0.717   0.014   0.269
B M  1JCKB                 21    0.121  24.172  -0.653   0.802   0.014   0.185
E G  1JCKB                 22    0.298  23.445  -0.749   0.802   0.014   0.185
B N  1JCKB                 23    0.068   9.999  -0.921   0.802   0.014   0.185
B M  1JCKB                 24    0.026   5.103   0.466   0.831   0.044   0.125
B K  1JCKB                 25    0.209  43.012   0.346   0.751   0.050   0.199
B Y  1JCKB                 26    0.230  49.087  -0.418   0.751   0.050   0.199
B L  1JCKB                 27    0.069  12.671   0.096   0.660   0.049   0.291
B Y  1JCKB                 28    0.094  20.152  -0.647   0.502   0.102   0.396
E D  1JCKB                 29    0.354  50.968  -0.696   0.191   0.086   0.723
E D  1JCKB                 30    0.479  69.009  -0.235   0.113   0.087   0.800
E H  1JCKB                 31    0.419  76.271  -0.205   0.052   0.084   0.864
E Y  1JCKB                 32    0.327  69.880  -0.269   0.021   0.279   0.699
B V  1JCKB                 33    0.123  18.844  -0.094   0.023   0.655   0.322
E S  1JCKB                 34    0.450  52.705   0.627   0.021   0.756   0.223
E A  1JCKB                 35    0.314  34.614  -0.566   0.023   0.655   0.322
E T  1JCKB                 36    0.554  76.798   0.451   0.023   0.655   0.322
E K  1JCKB                 37    0.586 120.458  -0.889   0.021   0.451   0.528
B V  1JCKB                 38    0.170  26.098   0.546   0.022   0.359   0.619
E K  1JCKB                 39    0.498 102.541  -0.016   0.022   0.552   0.426
E S  1JCKB                 40    0.456  53.455  -1.587   0.022   0.359   0.619
E V  1JCKB                 41    0.502  77.173  -1.639   0.020   0.205   0.775
E D  1JCKB                 42    0.583  83.967  -1.595   0.019   0.141   0.840
E K  1JCKB                 43    0.334  68.786   0.109   0.022   0.359   0.619
B F  1JCKB                 44    0.275  55.293  -0.300   0.004   0.514   0.481
B L  1JCKB                 45    0.170  31.072  -0.527   0.004   0.420   0.576
B A  1JCKB                 46    0.309  34.030  -1.279   0.004   0.420   0.576
B H  1JCKB                 47    0.197  35.907  -0.186   0.004   0.616   0.381
B D  1JCKB                 48    0.190  27.422   0.014   0.002   0.816   0.182
B L  1JCKB                 49    0.031   5.749   0.559   0.001   0.959   0.040
B I  1JCKB                 50    0.155  28.712   0.313   0.001   0.959   0.040
B Y  1JCKB                 51    0.047  10.129   1.031   0.002   0.816   0.182
E N  1JCKB                 52    0.260  38.020   0.580   0.004   0.514   0.481
B I  1JCKB                 53    0.200  37.037  -0.463   0.022   0.359   0.619
E N  1JCKB                 54    0.533  78.090  -0.886   0.021   0.279   0.699
E D  1JCKB                 55    0.337  48.533  -1.504   0.021   0.279   0.699
E K  1JCKB                 56    0.417  85.756  -0.435   0.021   0.279   0.699
E K  1JCKB                 57    0.563 115.850  -0.209   0.021   0.279   0.699
B L  1JCKB                 58    0.280  51.195  -0.758   0.020   0.205   0.775
E N  1JCKB                 59    0.581  85.014  -0.889   0.021   0.279   0.699
E N  1JCKB                 60    0.378  55.412  -0.598   0.022   0.359   0.619
B Y  1JCKB                 61    0.140  30.025  -0.031   0.074   0.484   0.442
E D  1JCKB                 62    0.354  50.983  -0.877   0.069   0.386   0.545
B K  1JCKB                 63    0.232  47.784   0.379   0.079   0.592   0.329
B V  1JCKB                 64    0.044   6.794   0.228   0.085   0.775   0.140
B K  1JCKB                 65    0.161  33.097   0.977   0.085   0.775   0.140
B T  1JCKB                 66    0.077  10.666  -0.639   0.087   0.683   0.230
E E  1JCKB                 67    0.269  47.012  -0.257   0.022   0.552   0.426
B L  1JCKB                 68    0.137  25.140  -1.026   0.022   0.359   0.619
E L  1JCKB                 69    0.369  67.491  -0.682   0.019   0.141   0.840
E N  1JCKB                 70    0.369  53.948  -0.514   0.018   0.047   0.935
E E  1JCKB                 71    0.553  96.522   0.260   0.858   0.002   0.139
E D  1JCKB                 72    0.546  78.621  -0.629   0.879   0.010   0.111
B L  1JCKB                 73    0.135  24.700   0.158   0.802   0.014   0.185
B A  1JCKB                 74    0.158  17.401  -0.644   0.802   0.014   0.185
E N  1JCKB                 75    0.618  90.519   0.814   0.694   0.003   0.303
E K  1JCKB                 76    0.430  88.410   1.047   0.600   0.003   0.397
B Y  1JCKB                 77    0.062  13.249  -0.034   0.406   0.004   0.590
E K  1JCKB                 78    0.519 106.820   0.743   0.181   0.016   0.803
E D  1JCKB                 79    0.735 105.914  -1.215   0.018   0.019   0.964
E E  1JCKB                 80    0.515  89.953   0.742   0.018   0.047   0.935
E V  1JCKB                 81    0.433  66.491   1.219   0.004   0.420   0.576
B V  1JCKB                 82    0.035   5.380   0.748   0.003   0.718   0.279
B D  1JCKB                 83    0.075  10.764  -0.424   0.003   0.718   0.279
B V  1JCKB                 84    0.029   4.473   0.263   0.001   0.900   0.099
B Y  1JCKB                 85    0.048  10.236   0.019   0.002   0.816   0.182
B G  1JCKB                 86    0.033   2.597  -1.427   0.004   0.616   0.381
B S  1JCKB                 87    0.043   5.004   0.172   0.004   0.514   0.481
B N  1JCKB                 88    0.168  24.668  -1.214   0.021   0.451   0.528
B Y  1JCKB                 89    0.127  27.247  -0.950   0.021   0.279   0.699
B Y  1JCKB                 90    0.260  55.647  -1.679   0.020   0.205   0.775
B V  1JCKB                 91    0.142  21.872  -1.782   0.021   0.279   0.699
E N  1JCKB                 92    0.304  44.447  -0.826   0.021   0.279   0.699
B C  1JCKB                 93    0.077  10.825  -1.583   0.066   0.296   0.638
E Y  1JCKB                 94    0.381  81.505  -1.033   0.066   0.296   0.638
E F  1JCKB                 95    0.315  63.220  -1.386   0.064   0.216   0.721
E S  1JCKB                 96    0.325  38.149  -1.186   0.064   0.216   0.721
E S  1JCKB                 97    0.463  54.299  -1.706   0.118   0.150   0.732
E K  1JCKB                 98    0.555 114.163  -1.517   0.118   0.150   0.732
E D  1JCKB                 99    0.469  67.568  -1.340   0.125   0.227   0.648
E N  1JCKB                100    0.412  60.375  -1.497   0.135   0.317   0.548
E V  1JCKB                101    0.541  83.167  -1.853   0.066   0.296   0.638
E G  1JCKB                102    0.484  38.107  -2.329   0.064   0.216   0.721
E K  1JCKB                103    0.509 104.784  -1.503   0.064   0.216   0.721
E V  1JCKB                104    0.388  59.651  -0.955   0.064   0.216   0.721
E T  1JCKB                105    0.502  69.697  -1.502   0.020   0.205   0.775
E S  1JCKB                106    0.575  67.413  -1.139   0.018   0.088   0.893
E G  1JCKB                107    0.544  42.844  -0.407   0.018   0.047   0.935
E K  1JCKB                108    0.284  58.378   0.144   0.005   0.262   0.733
B T  1JCKB                109    0.190  26.325   0.600   0.003   0.718   0.279
B C  1JCKB                110    0.051   7.160   0.334   0.001   0.900   0.099
B M  1JCKB                111    0.044   8.744  -0.693   0.001   0.900   0.099
B Y  1JCKB                112    0.073  15.664  -0.719   0.002   0.816   0.182
B G  1JCKB                113    0.068   5.336  -1.971   0.004   0.420   0.576
B G  1JCKB                114    0.100   7.846  -2.020   0.021   0.451   0.528
B I  1JCKB                115    0.056  10.434  -0.162   0.003   0.718   0.279
B T  1JCKB                116    0.186  25.770   0.882   0.023   0.655   0.322
E K  1JCKB                117    0.349  71.769   0.506   0.021   0.451   0.528
E H  1JCKB                118    0.398  72.469  -0.051   0.021   0.279   0.699
E E  1JCKB                119    0.596 104.121  -0.791   0.056   0.142   0.802
E G  1JCKB                120    0.550  43.301  -1.334   0.056   0.142   0.802
E N  1JCKB                121    0.522  76.348  -0.178   0.069   0.386   0.545
E H  1JCKB                122    0.486  88.403   0.164   0.074   0.484   0.442
E F  1JCKB                123    0.283  56.838  -0.547   0.069   0.386   0.545
E D  1JCKB                124    0.517  74.428  -1.516   0.064   0.216   0.721
E N  1JCKB                125    0.550  80.447  -0.726   0.056   0.142   0.802
E G  1JCKB                126    0.585  46.079  -1.563   0.020   0.205   0.775
E N  1JCKB                127    0.389  56.979  -0.791   0.021   0.279   0.699
E L  1JCKB                128    0.416  76.115  -1.623   0.021   0.279   0.699
B Q  1JCKB                129    0.200  35.684  -0.141   0.004   0.420   0.576
E N  1JCKB                130    0.373  54.534  -0.155   0.004   0.616   0.381
B V  1JCKB                131    0.021   3.228   0.865   0.003   0.718   0.279
B L  1JCKB                132    0.129  23.528  -0.733   0.002   0.816   0.182
B I  1JCKB                133    0.039   7.178  -0.888   0.002   0.816   0.182
E R  1JCKB                134    0.258  59.174   0.136   0.001   0.900   0.099
B V  1JCKB                135    0.046   7.009  -0.393   0.002   0.816   0.182
E Y  1JCKB                136    0.294  62.721  -0.357   0.004   0.616   0.381
E E  1JCKB                137    0.357  62.298  -0.156   0.004   0.420   0.576
E N  1JCKB                138    0.624  91.383  -0.496   0.004   0.085   0.910
E K  1JCKB                139    0.518 106.511  -1.309   0.004   0.138   0.858
E R  1JCKB                140    0.524 119.950   0.086   0.003   0.718   0.279
E N  1JCKB                141    0.412  60.273   0.143   0.002   0.816   0.182
E T  1JCKB                142    0.330  45.840   0.421   0.004   0.616   0.381
E I  1JCKB                143    0.362  66.933  -0.224   0.004   0.616   0.381
E S  1JCKB                144    0.409  47.947  -1.366   0.004   0.616   0.381
B F  1JCKB                145    0.232  46.582  -0.518   0.004   0.616   0.381
E E  1JCKB                146    0.358  62.595   0.263   0.002   0.816   0.182
B V  1JCKB                147    0.058   8.976   0.300   0.011   0.918   0.071
E Q  1JCKB                148    0.374  66.779   0.571   0.001   0.900   0.099
B T  1JCKB                149    0.081  11.165  -0.440   0.003   0.718   0.279
E D  1JCKB                150    0.586  84.500   0.276   0.005   0.262   0.733
E K  1JCKB                151    0.419  86.085   0.436   0.018   0.088   0.893
E K  1JCKB                152    0.539 110.893   0.158   0.020   0.205   0.775
E S  1JCKB                153    0.477  55.881   1.042   0.023   0.655   0.322
B V  1JCKB                154    0.066  10.175   0.525   0.021   0.756   0.223
B T  1JCKB                155    0.170  23.524   0.939   0.085   0.775   0.140
B A  1JCKB                156    0.062   6.821   0.128   0.172   0.690   0.138
B Q  1JCKB                157    0.235  42.060   0.761   0.273   0.587   0.140
B E  1JCKB                158    0.188  32.809  -0.263   0.487   0.370   0.144
B L  1JCKB                159    0.065  11.810   0.684   0.649   0.163   0.188
B D  1JCKB                160    0.089  12.767   0.270   0.751   0.050   0.199
B I  1JCKB                161    0.141  26.122   0.685   0.879   0.010   0.111
E K  1JCKB                162    0.296  60.949   1.224   0.938   0.007   0.055
B A  1JCKB                163    0.032   3.559   0.228   0.938   0.007   0.055
B R  1JCKB                164    0.144  33.022   0.931   0.975   0.003   0.022
E N  1JCKB                165    0.379  55.486   1.105   0.975   0.003   0.022
B F  1JCKB                166    0.229  46.061   1.412   0.975   0.003   0.022
B L  1JCKB                167    0.026   4.761   0.274   0.938   0.007   0.055
B I  1JCKB                168    0.248  45.899   0.644   0.938   0.007   0.055
E N  1JCKB                169    0.630  92.203   0.389   0.879   0.010   0.111
E K  1JCKB                170    0.340  69.979   0.496   0.717   0.014   0.269
B K  1JCKB                171    0.206  42.312   0.676   0.561   0.047   0.393
E N  1JCKB                172    0.390  57.155   0.188   0.428   0.171   0.402
B L  1JCKB                173    0.147  27.007   0.146   0.231   0.330   0.439
B Y  1JCKB                174    0.137  29.277  -0.064   0.135   0.317   0.548
E E  1JCKB                175    0.423  73.898  -0.254   0.064   0.216   0.721
E F  1JCKB                176    0.352  70.606  -0.957   0.052   0.084   0.864
E N  1JCKB                177    0.706 103.314  -1.412   0.018   0.019   0.964
E S  1JCKB                178    0.690  80.821  -2.085   0.018   0.019   0.964
E S  1JCKB                179    0.318  37.281  -1.834   0.018   0.019   0.964
E P  1JCKB                180    0.517  73.348   0.387   0.019   0.141   0.840
B Y  1JCKB                181    0.151  32.311   0.016   0.004   0.420   0.576
E E  1JCKB                182    0.378  66.106  -0.171   0.004   0.616   0.381
E T  1JCKB                183    0.401  55.619   0.755   0.003   0.718   0.279
B G  1JCKB                184    0.055   4.321  -0.574   0.002   0.816   0.182
B Y  1JCKB                185    0.180  38.423   1.256   0.001   0.959   0.040
B I  1JCKB                186    0.030   5.550   0.277   0.001   0.959   0.040
B K  1JCKB                187    0.136  28.057   0.652   0.000   0.983   0.017
B F  1JCKB                188    0.028   5.559   0.706   0.001   0.959   0.040
B I  1JCKB                189    0.139  25.752   0.666   0.001   0.959   0.040
B E  1JCKB                190    0.132  23.095  -0.428   0.003   0.718   0.279
E S  1JCKB                191    0.568  66.581  -0.179   0.004   0.197   0.799
E N  1JCKB                192    0.685 100.225  -0.973   0.005   0.015   0.979
E G  1JCKB                193    0.686  53.980  -1.122   0.003   0.003   0.994
E N  1JCKB                194    0.566  82.906   0.221   0.004   0.085   0.910
E T  1JCKB                195    0.350  48.545   0.947   0.003   0.718   0.279
B F  1JCKB                196    0.123  24.706   0.946   0.001   0.959   0.040
B W  1JCKB                197    0.147  35.426   0.016   0.001   0.959   0.040
B Y  1JCKB                198    0.045   9.723   0.837   0.001   0.959   0.040
B D  1JCKB                199    0.059   8.574  -1.284   0.002   0.816   0.182
B M  1JCKB                200    0.031   6.223   0.219   0.022   0.552   0.426
B M  1JCKB                201    0.061  12.166   0.058   0.004   0.514   0.481
B P  1JCKB                202    0.171  24.222  -0.688   0.004   0.420   0.576
B A  1JCKB                203    0.228  25.170   0.199   0.018   0.088   0.893
E P  1JCKB                204    0.618  87.623  -1.122   0.018   0.019   0.964
E G  1JCKB                205    0.646  50.848  -2.866   0.016   0.005   0.979
E D  1JCKB                206    0.569  81.950  -1.687   0.018   0.019   0.964
E K  1JCKB                207    0.389  79.997  -1.339   0.053   0.043   0.903
B F  1JCKB                208    0.228  45.699  -1.462   0.053   0.043   0.903
E D  1JCKB                209    0.445  64.052  -0.867   0.113   0.043   0.844
E Q  1JCKB                210    0.352  62.831  -0.782   0.278   0.093   0.628
E S  1JCKB                211    0.373  43.727  -0.759   0.321   0.252   0.427
E K  1JCKB                212    0.356  73.147   0.159   0.352   0.332   0.316
B Y  1JCKB                213    0.107  22.759   0.213   0.352   0.332   0.316
B L  1JCKB                214    0.071  13.018  -0.302   0.373   0.401   0.226
E M  1JCKB                215    0.298  59.530   0.731   0.252   0.423   0.325
B I  1JCKB                216    0.173  31.987   0.038   0.268   0.505   0.227
B Y  1JCKB                217    0.125  26.734  -0.049   0.231   0.330   0.439
E K  1JCKB                218    0.519 106.717  -0.314   0.118   0.150   0.732
E D  1JCKB                219    0.507  73.030  -0.965   0.053   0.043   0.903
E N  1JCKB                220    0.463  67.812  -1.331   0.018   0.047   0.935
E K  1JCKB                221    0.459  94.499   0.166   0.019   0.141   0.840
E M  1JCKB                222    0.344  68.834   0.197   0.021   0.451   0.528
B V  1JCKB                223    0.110  16.830   0.563   0.023   0.655   0.322
E D  1JCKB                224    0.509  73.347   0.379   0.021   0.451   0.528
B S  1JCKB                225    0.240  28.105  -1.160   0.021   0.279   0.699
E K  1JCKB                226    0.608 125.045  -0.439   0.005   0.336   0.660
E S  1JCKB                227    0.453  53.103   0.965   0.004   0.420   0.576
B V  1JCKB                228    0.097  14.986   0.476   0.003   0.718   0.279
B K  1JCKB                229    0.223  45.912   0.497   0.001   0.900   0.099
B I  1JCKB                230    0.049   9.046   0.690   0.001   0.900   0.099
B E  1JCKB                231    0.110  19.234  -0.053   0.001   0.959   0.040
B V  1JCKB                232    0.027   4.227   0.808   0.001   0.959   0.040
B H  1JCKB                233    0.146  26.630   0.558   0.001   0.959   0.040
B L  1JCKB                234    0.035   6.335   0.560   0.001   0.959   0.040
B T  1JCKB                235    0.167  23.163  -0.031   0.001   0.959   0.040
E T  1JCKB                236    0.296  41.111   0.398   0.001   0.900   0.099
E K  1JCKB                237    0.362  74.402  -0.228   0.004   0.616   0.381
E N  1JCKB                238    0.649  95.058  -1.345   0.019   0.141   0.840
E G  1JCKB                239    0.880  69.272  -1.904   0.006   0.001   0.993

Explain the output.  Go back.
```

---
